# Modulating Target Protein Biology Through the Re-mapping of Conformational Distributions Using Small Molecules

**DOI:** 10.3389/fchem.2021.668186

**Published:** 2021-05-04

**Authors:** Alastair D. G. Lawson, Malcolm MacCoss, Dominique L. Baeten, Alex Macpherson, Jiye Shi, Alistair J. Henry

**Affiliations:** ^1^UCB Pharma, Slough, United Kingdom; ^2^Bohicket Consulting LLC, Seabrook Island, SC, United States

**Keywords:** protein-protein interactions, conformational sampling, conformational distributions, conformer, allosteric, small molecule, drug discovery, molecular dynamics

## Abstract

Over the last 10 years considerable progress has been made in the application of small molecules to modulating protein-protein interactions (PPIs), and the navigation from “undruggable” to a host of candidate molecules in clinical trials has been well-charted in recent, comprehensive reviews. Structure-based design has played an important role in this scientific journey, with three dimensional structures guiding medicinal chemistry efforts. However, the importance of two additional dimensions: movement and time is only now being realised, as increasing computing power, closely aligned with wet lab validation, is applied to the challenge. Protein dynamics are fundamental to biology and disease, and application to PPI drug discovery has massively widened the scope for new chemical entities to influence function from allosteric, and previously unreported, sites. In this forward-looking perspective we highlight exciting, new opportunities for small molecules to modulate disease biology, by adjusting the frequency profile of natural conformational sampling, through the stabilisation of clinically desired conformers of target proteins.

## Introduction

Recent reviews (Lu et al., 2020; Serapian and Colombo, 2020) have skilfully and comprehensively described progress in the development of PPI small molecule modulators, building on the work of pioneers such as Jim Wells in the early 2000s (Arkin and Wells, 2004; Hardy and Wells, 2004), and with the approval of Venetoclax (ABT-199) in 2016, a trail has now been blazed. Venetoclax was the first BCL-2-selective BH3-mimetic to achieve FDA approval for Chronic Lymphocytic Leukaemia (CLL), and thus represents a milestone in small molecules targeting PPIs (Roberts and Huang, 2017). Structural Biology has been an important companion on this journey, as three-dimensional structures of proteins continue to inform computer-aided drug design (CADD). However, such images, although visually compelling, do not give us the full picture, often hiding opportunities for allosteric inhibition and stabilisation (Ni et al., 2019), because they lack two vital components: movement and time.

This spatial-temporal relationship is called protein dynamics and is increasingly seen as an important facet of normal biology and pathophysiology (Bu and Callaway, 2011), complementing genetic variation and post-translational modification.

In order to understand the concept of protein dynamics, the importance of conformational sampling of different structural states in biology and disease, and how we can intervene clinically with novel therapeutics to restore normal function, an analogy may be helpful. Let us consider a target protein as a bascule, or lifting bridge, over a canal!

## The Canal Bridge

The bridge has two useful functions: enabling road traffic and barge traffic and must adopt two different conformations to service both but at different times. The bridge thus creates an either/or solution using the same structural elements. The distribution of angles of the drawbridge ranges from 0° (fully down and open to road traffic) to 90° (up and open to barges), with 1–89° angles visited during transition. These are the main structural conformations the bridge samples, but in addition there is a time element demonstrating that in normal operation it spends 97% of the time at 0° (allowing the greater volume of road traffic to flow freely), 2% at 90° (allowing the passage of occasional barges) and 1% in the range 1–89° moving between the normal operational states. With the bridge fully functional and free to sample all conformations with appropriate frequency, road hauliers and barge masters are both well and differentially served.

As the bridge spends the majority of its time at 0°, satellite photographs are likely to show it in this closed state and give no clue about its open 90° state. Not only is the 0° state the most likely to be observed, it is also the least energetically costly and will require input of energy to perturb. So it is with crystal structures; there is no evidence that other conformations are possible; we thus have a narrow view and limited options for drug targeting.

In contrast, the 90° state can be thought of as a metastable, higher energy state, that would fall to 0° in the absence of energy input but is still fairly stable once achieved. A hydraulic stepping motor is used to input energy between 0 and 90°, and achieves the desired lift in three stages, briefly pausing at 30 and 60° for re-priming to accommodate ram extensions. These two intermediate states at 30 and 60° can be seen as islands of high energy, sampled during every ascent, and descent but with short residence times.

If there is a problem with the hydraulic lifting mechanism, either a design fault (equivalent to a genetic defect) or a maintenance issue (representing disease), the ability of the bridge to sample the required conformational states, with the appropriate frequency to function effectively, will be compromised.

For example, if there is a problem with the hydraulic motor, the bridge may be unable to lift beyond 60° at every attempt, preventing the free passage of barges on the canal. Barge masters face delays and may use the steel bows of their barges to nudge the bridge up from 60° to enable access, which may be thought of as induced fit!

Conversely if the dump valve is mal-functioning, the bridge may be unable to close fully at every attempt, resting at the 30° point and blocking the road. Road hauliers face detours, and joy riders in stolen cars compound the problem by attempting to jump the gap, crashing into the canal and filling hospital beds, relating to disease pathology.

The distribution of potential angles sampled by the faulty bridge is the same, but the distribution of time spent in each state is different. For example with a malfunction rate of 90% the time distribution is:

**Table:** 

	**0 degrees %**	**1–89 degrees %**	**90 degrees %**
Normal operation	97	1	2
Fault with hydraulic motor	97	2.8	0.2
Fault with dump valve	9.7	88.3	2
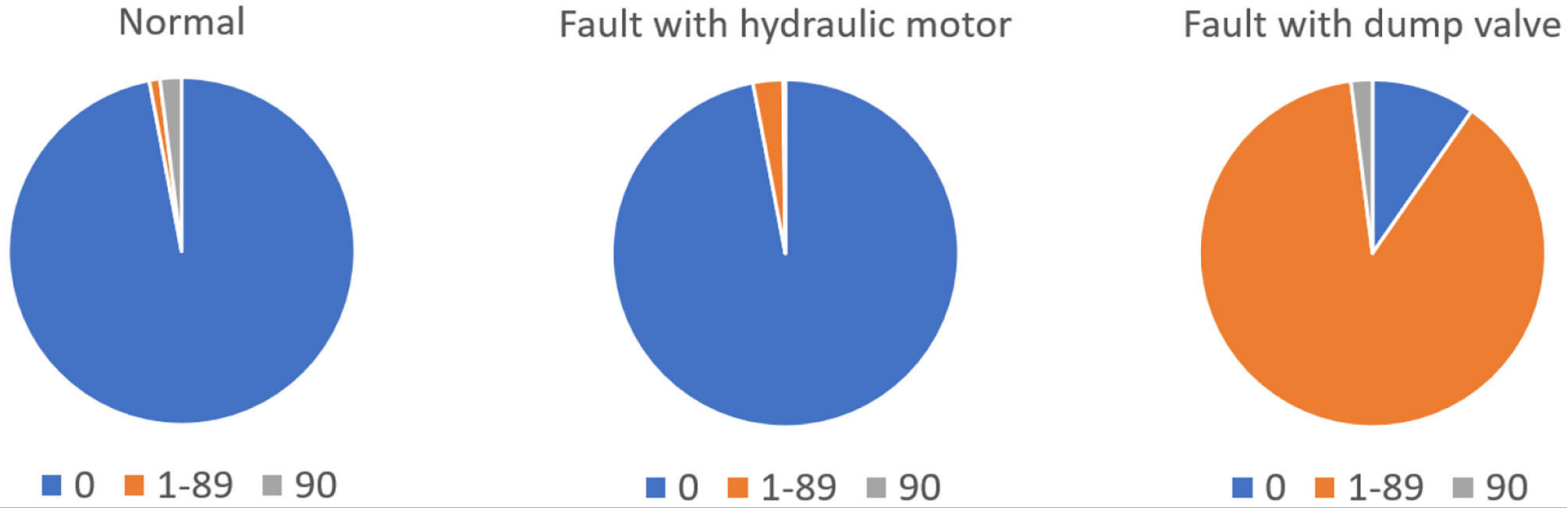			

The pie chart illustrates the percentage of time the bridge spends sampling different angles (0, 1–89, and 90°).

Of course, with the bridge malfunctioning, it may be decided that road transport is more important to the economy and should be prioritised, in which case the bridge could be held in the 0° state and the hydraulic lifting mechanism overcome; this would equate to a blocking antibody approach orthosterically preventing raising by placing a heavy weight at the end of the bridge.

Conversely, should barge traffic be prioritised, the bridge could be tagged for demolition, which would be more like a targeted protein degradation (TPD) approach, where a small molecule brings a target into close proximity with an E3 ligase, leading to ubiquitination of the target and its subsequent destruction.

An elegant and desirable solution to the problem in each case would be to place small wedges in at the hinge of the bridge to facilitate either complete raising or lowering. The wedges can be thought of as small molecules reversibly re-mapping the conformational sampling of target proteins from allosteric sites according to their angles.

## Learning From the Professionals

Antibodies have a few 100 million years start on us in targeting PPIs, and hence are well-placed to be our allies in conformational definition of target proteins. We can also meaningfully consider how the immune system has evolved to create extraordinary diversity of binding sites with positive selection of hits and analogues based solely on affinity. Nature worked out that the PPI problem was too difficult to solve by design in time and that selection from ultra-large, constantly replenished libraries of variable region sequences, which are linked to their amplifiable, genetic blueprints, was the practical solution.

In addition, antibodies that modify function can teach us about how they are doing it, and while many will simply be acting orthosterically, some will be binding to allosteric sites and exerting their influence at distal interfaces (Staus et al., 2016). The latter are of particular interest, as in many cases they will be stabilising conformations of interest which are not represented in the Protein Data Bank (PDB). A particularly striking example was provided from the binding of the Fab fragment of an antibody to IgE, an important target in asthma (Drinkwater et al., 2014). This showed the most potent, neutralising Fab molecules stabilising an extended, linear conformation of IgE, very different from the bent form represented in crystal structures. In this case the antibody provided useful wet lab validation for a conformation which had been predicted by molecular dynamics simulations but thought likely to be only very rarely sampled.

Function-modifying antibodies, which are stabilising druggable conformations, can be used as scaffolding to define and present the target in an energy-minimised state, just as scaffolding could be used by a maintenance crew working on the canal bridge to support the 30 or 60° states when checking the wedges. With the target protein frozen in time, hidden pockets only available as the protein passes naturally through transiently sampled conformers can be revealed. These allosteric binding sites are likely to be less well-conserved than orthosteric sites and can be exploited in screens utilising such antibody/target protein complexes, to enable weakly binding, but possibly more specific, chemical starting points to gain a foothold. Following elaboration, binding of the compound becomes robust enough for the antibody scaffolding to be removed. As the antibody has already validated the clinical desirability of the specific target conformation, if the compound can faithfully reproduce this same conformation it can be expected to achieve similar biology, considerably reducing biological risk in small molecule drug discovery (Lawson, 2012). Use of an antibody in this context increases the probability of discovering small molecules that can stabilise interesting conformers of target proteins but does not guarantee that each small molecule that binds to an antibody-stabilised state will be able to define that conformer once the antibody has been removed.

## Drug Discovery

The study of molecular dynamics aims to address important gaps in our knowledge to predict possible conformations, reveal cryptic binding sites and support drug discovery through computational modelling (Kuzmanic et al., 2020). With ever increasing amounts of computing power being applied to predicting protein dynamics, we are seeing accuracy increasing, and no doubt machine-learning and artificial intelligence will find their place (Greener and Sternberg, 2018). But such advances are only as good as the quality and quantity of the input data and are thus influenced and possibly curtailed by the bias of previous experience (Rodrigues, 2020). In addition, the timeframe for large protein movements is greater than, and not always compatible with, the computationally power-hungry microsecond to millisecond timeframes needed for meaningful molecular dynamics simulations (Cossins and Lawson, 2015), leading to over emphasis on local effects with potentially important and advantageously druggable conformations remaining unexplored.

It is thus vitally important for such *in silico* predictions to not become self-fulfilling prophesies, but that they are tested and validated in the real world. Wet lab techniques such as Double Electron-electron Resonance (DEER) are particularly useful in this regard, providing distance measurements which can confirm expected conformers or indicate new states. Existing crystal structures can be adjusted to create working models of target proteins in biologically relevant conformations. Even lowly populated states can be revealed by DEER, such as the distorted trimer of TNF, which had a natural sampling frequency of just 6% (Carrington et al., 2017), but had been predicted by molecular dynamics simulations. This naturally sampled deviation from trimerous symmetry proved to be druggable, with allosteric, orally available small molecules, which stabilise the distorted trimer and inhibit signalling through TNFR1, being discovered from an original fragment hit (O'Connell et al., 2019; McMillan et al., 2021). TNF has long been successfully targeted by biologics acting orthosterically (Lim et al., 2018), and the demonstration that equivalent biology can be achieved by small molecules binding allosterically represents a quantum step forward in tackling high affinity (pM) PPIs featuring large surface areas of engagement. Antibody definition of the specifically distorted state has also provided an extremely useful target engagement biomarker for the small molecule (Lightwood et al., 2021), and offers scope to increase intellectual property rights beyond just the chemical matter.

Compounds working in this way bind during the brief period when the protein is sampling the desired state to stabilise this conformation and re-equilibrate the biology. They thus tend to have slow apparent association rates, while they wait for the protein to sample the correct orientation with limited temporal accessibility, and thus benefit from being cell-penetrant, so that the protein is secreted already in a small molecule-normalised and clinically sought state. In addition, compounds with this mechanism of action have slow dissociation rates, and just like the wedges acting at the hinge in the canal bridge, need to be of rigid, non-flexible construction, so that occupancy of their binding site is enough to translate directly to the desired effect resulting from their binding. Wedges that bend or become distorted when used in the hinge can create a situation of 100% occupancy, but with <100% Emax in functional assays, as the compound-bound protein can occasionally still move sufficiently to sample clinically undesirable conformers (Lawson et al., 2018).

While the illustrations so far have focused primarily on inhibiting PPIs, similar concepts can be applied to promoting desired and naturally occurring PPIs, as in Targeted Protein Degradation, for example. Small molecules may be used as bi-specific reagents (PROTACs) or as molecular glues, and as the target-preferred ligase interactions for the protein-protein complex pre-exist, the energy barrier to agonism may well be easier to overcome with compounds featuring only modest affinity (Schreiber, 2019). In addition, compounds could be used to stabilise one partner in a favourable conformation to augment binding.

## Sources of Chemical Matter

During the 1990s constraints on the sampling of chemical diversity, imposed by the archiving and screening logistics associated with high throughput screening (HTS), were addressed by the introduction of libraries featuring low molecular weight fragments capable of representing broader chemical space than conventional compound decks (Hajduk et al., 1997). While the approach has been successful, in that a number of drugs arising from fragment-based discovery have been approved (Valenti et al., 2019; Osborne et al., 2020), experience also shows that it can be a long haul to go from analogues of a millimolar affinity small fragment hit to first get on scale in an activity assay and then to build in sufficient potency for the compound to be considered a lead, particularly for PPI targets, even with structural data to guide the design.

Application of molecular dynamics simulations to provide flexible docking for virtual screening has enabled large libraries of fragments and compounds to be screened and ranked *in silico*, so that virtual hits can be more accurately prioritised before their binding is confirmed in the wet lab (Bissaro et al., 2020; Lin et al., 2020). Accuracy with this approach is closely linked to increasing computing power, and the method is perhaps most useful in providing valuable information about both druggable sites and concomitant chemical matter.

DNA-encoded chemical libraries (Brenner and Lerner, 1992; Neri and Lerner, 2018; Madsen et al., 2020) offer unparalleled diversity and fast hit identification, using technology closely related to antibody phage library panning (Smith, 1985), in that the instructions for synthesis are physically linked to the compound. Early application to the LFA-1/ICAM PPI demonstrated the potential (Kollmann et al., 2014). Nanomolar affinities with commendable solubility and developability can be obtained from hits “straight out of the box,” and although attrition is seen at the re-synthesis and DNA-removal steps, the technology is already fulfilling its promise. In many ways this option would seem to offer the best of both worlds combining strong representation of diverse chemical space with affinity-based one pot selection of hits; the parallels with natural antibody generation are striking. Second round screening in carefully designed activity assays, followed by tertiary screening on cells, can relatively quickly define useful compounds ready to enter the hit-to-lead stage. Libraries of this size are likely to be needed to take full advantage of the new biological space becoming available for PPIs, with traditional chemical collections, which were predominantly designed to service enzyme pockets, sometimes giving very few hits in this area of space.

## Discussion

Recent papers show that PPI targets, for long considered the preserve of biologicals, are succumbing to small molecules which bind allosterically at relatively protected, and often less assiduously conserved, allosteric sites. The compounds stabilise naturally sampled conformations of protein targets, which are either incompatible with binding to partner proteins, or promote clinically favourable interactions. Thus, the adage that such interactions were undruggable with small molecules can be overturned through imaginative thinking, advances in technology and by re-mapping the conformational distributions of proteins. By tapping into the natural fine tuning of conformational state space, which underpins biological function and regulation (Schmid and Hugel, 2020), we can advantageously engage small molecules to modulate PPIs in precise and subtle ways.

For all the intellectual elegance of *in silico* methods and the seductive power of today's technology, serendipity and open minds will remain essential components of innovative drug discovery, and we as an industry should remember that patients don't care how their drugs were discovered; they just want them to be effective and safe, and they want them now.

## Data Availability Statement

The original contributions generated for the study are included in the article/supplementary material, further inquiries can be directed to the corresponding author/s.

## Author Contributions

All authors contributed to scientific concepts and writing the manuscript.

## Conflict of Interest

AL, DB, AM, JS and AH are employed by the company UCB Pharma. MM was employed by Bohicket Consulting LLC.
